# Single Tumor Cells With Epithelial-Like Morphology Are Associated With Breast Cancer Metastasis

**DOI:** 10.3389/fonc.2020.00050

**Published:** 2020-02-19

**Authors:** Liubov A. Tashireva, Marina V. Zavyalova, Olga E. Savelieva, Tatyana S. Gerashchenko, Evgeniya V. Kaigorodova, Evgeny V. Denisov, Vladimir M. Perelmuter

**Affiliations:** ^1^Department of General and Molecular Pathology, Cancer Research Institute, Tomsk National Research Medical Center, Tomsk, Russia; ^2^Department of Pathological Anatomy, Siberian State Medical University, Tomsk, Russia; ^3^Laboratory of Cancer Progression Biology, Cancer Research Institute, Tomsk National Research Medical Center, Tomsk, Russia; ^4^Department of Biochemistry and Molecular Biology, Siberian State Medical University, Tomsk, Russia; ^5^Department of Organic Chemistry, Tomsk State University, Tomsk, Russia

**Keywords:** single tumor cells, breast cancer, EMT, stem cell, distant metastasis

## Abstract

**Introduction:** The identification of tumor cells that can be potential metastatic seeds would reach two key aims—prognosis of metastasis risk and appointment of the optimal adjuvant therapy to prevent metastatic disease. Single tumor cells (STCs) located out of multicellular structures can most likely demonstrate features that are needed to initiate metastasis.

**Methods:** One-hundred-and-thirty-five patients with invasive breast carcinoma of no special type have been enrolled. Molecular subtypes of breast cancer were categorized according to St. Gallen recommendations. Hematoxylin and eosin staining was used to identify STCs with epithelial-like morphology (eSTCs) in breast tumors. Immunofluorescence staining was applied to evaluate stemness and epithelial–mesenchymal transition (EMT) in STCs. The correlation between STCs and recurrence and metastasis-free survival (MFS) was performed using the Kaplan–Meier method and the log-rank test.

**Results:** Distant metastasis was more frequent in eSTC-positive than eSTC-negative patients (28.0% vs. 9.4%, *p* = 0.007). When tumor types were analyzed separately, distant metastasis tended to be more frequent in eSTC-positive than eSTC-negative patients for HER2-positive cancer [75.0% (3/4) vs. 12.5% (1/8), *p* = 0.066]. In luminal A [22.7% (5/22) vs. 10.0% (3/30), *p* = 0.259], luminal B [21.1% (4/19) vs. 6.7% (2/30), *p* = 0.189], and triple-negative [40.0% (2/5) vs. 11.8% (2/17), *p* = 0.209] cancers, distance metastasis was not associated with eSTCs. Median MFS was not reached in eSTC-positive and eSTC-negative patients. eSTC-positive patients had a higher risk of breast cancer metastasis [hazard ratio (HR) 3.57, 95% confidence interval (CI): 1.46–8.71; *p* = 0.001]. When tumor types were analyzed separately, a higher risk of breast cancer metastasis occurred only in HER2-positive patients (HR 8.49, 95% CI: 1.29–55.59; *p* = 0.016). Immunofluorescence analysis revealed mesenchymal-like STCs (mSTCs) and inter- and intra-tumor heterogeneity in STCs. There were breast tumors with either eSTCs or mSTCs and tumors with both types of STCs. Both eSTCs and mSTCs were represented by cells with different stem and/or EMT phenotypes.

**Conclusions:** STCs with epithelial-like morphology contribute to breast cancer metastasis and represent an attractive model for studying mechanisms of metastatic seeding. The assessment of STCs in histological sections of breast tumors can be a simple and effective method for the prediction of metastasis risk.

## Introduction

The prediction of tumor progression risk, including lymph node and distant metastasis, remains one of the most important problems in modern oncology. Metastasis can occur not only by single tumor cells (STCs) but also by tumor cell clusters (1). In the past, metastasis was thought to occur by retention of metastatic cells in the capillary system of the first parenchymatous organ encountered (2). This hypothesis was subsequently dismissed as metastatic cells were shown to reach the vasculature of all organs (2). Moreover, clusters of tumor cells are able to pass through capillary-sized vessels (3). Recent findings give further support to the “seed and soil” hypothesis (2) that focuses on the dissemination of STCs.

STCs are a manifestation of intratumor morphological heterogeneity and most likely result from multicellular tumor structures through cancer invasion. In the last years, the appearance of STCs is described as tumor budding in the invasive front (4).

Invasive carcinoma of no special type (IC NST), the most common form of breast cancer (5), is highly heterogeneous in the morphological pattern. Previously, we showed that breast tumor cells can be either single, arranged in small (discrete) groups, or arranged in more complex structures (tubular, alveolar, solid, and trabecular) (6). In addition, we suggested that the intratumor morphological heterogeneity in breast cancer is a result of the unfolding of the invasion program during which epithelial–mesenchymal transition (EMT) leads to significant morphogenetic changes in the tumor landscape: from tubular structures that are close to normal mammary ducts to discrete groups of tumor cells demonstrating a strongly pronounced mesenchymal phenotype (7). The recent study hypothesized that the intratumor morphological heterogeneity can be an attractive model for studying the mechanisms of collective cell invasion (by focusing on solid and trabecular structures) and individual cell invasion (by focusing on discrete groups, namely, STCs) (8).

According to current understanding, STCs may be in a quiescent state or invade by mesenchymal, amoeboid, and hybrid mesenchymal–amoeboid motion (9, 10). The definition of these STC states could be an effective tool for studying the mechanisms of cancer invasion and intravasation and would help to predict metastasis risk.

Thus, the aim of this study was to assess morphological and phenotypical heterogeneity of STCs and their prognostic significance in breast cancer patients.

## Materials and Methods

### Patients and Specimens

The retrospective study included 135 patients with IC NST (stage I-IIIC, T_1−4_N_0−3_M_0_) who were treated in the Cancer Research Institute, Tomsk NRMC between 2008 and 2015 (Table 1). The median age was 55 years (range: 29–85 years). All cases were reexamined, and IC NST was diagnosed and staged according to the World Health Organization's recommendations (5). Patients had not received neoadjuvant chemotherapy and were monitored using computed tomography (CT) scan every 6 months to identify metastatic lesions. Recurrence- and metastasis-free survival (RFS and MFS) was defined as the time window spanning between the diagnosis and the detection of the first recurrence or metastatic lesion on imaging or patient death, whichever occurred first.

**Table 1:** Clinicopathological characteristics of breast cancer patients.

**Characteristics**		**No eSTCs, % (*n*)**	**Yes eSTCs, % (*n*)**	***p***
Postoperative treatment	Adjuvant chemotherapy: CMF, FAC, CAX	80 (68)	82 (41)	NS
	Antiestrogen therapy with tamoxifen	20 (17)	18 (9)	NS
Age	<35 years	35 (30)	26 (13)	NS
	35–50 years	22 (19)	36 (18)	NS
	>50 years	43 (36)	38 (19)	NS
Menopausal status	Premenopausal	33 (28)	36 (18)	NS
	Postmenopausal	67 (57)	64 (32)	NS
Stage	I (T_1_N_0_M_0_)	28 (24)	16 (8)	NS
	IIA (T_0−1_N_1_M_0_, T_2_N_0_M_0_)	39 (33)	32 (16)	NS
	IIB (T_2_N_1_M_0_, T_3_N_0_M_0_)	13 (11)	14 (7)	NS
	IIIA (T_0−2_N_2_M_0_, T_3_N_1−2_M_0_)	14 (12)	30 (15)	NS
	IIIB (T_4_N_0−2_M_0_)	1 (1)	0 (0)	NS
	IIIC (T_1−4_N_3_M_0_)	5 (4)	8 (4)	NS
Grade	I	7 (6)	10 (5)	NS
	II	74 (63)	80 (40)	NS
	III	19 (16)	10 (5)	NS
Tumor size	<2 cm	55 (47)	30 (15)	0.007
	2–5 cm	44 (37)	62 (31)	0.049
	>5 cm	1 (1)	8 (4)	NS
Molecular subtype	Luminal A	35 (30)	44 (22)	NS
	Luminal B	35 (30)	38 (19)	NS
	Triple-negative	20 (17)	10 (5)	NS
	HER2-positive	10 (8)	8 (4)	NS
Estrogen receptors	Positive	75 (64)	86 (43)	NS
	Negative	25 (20)	14 (7)	NS
Progesterone receptors	Positive	62 (53)	64 (32)	NS
	Negative	38 (32)	36 (18)	NS
HER2	Positive	20 (17)	26 (13)	NS
	Negative	80 (68)	74 (37)	NS
Ki-67	Expression <20%	36 (31)	48 (24)	NS
	Expression > 20%	64 (54)	52 (26)	NS

*CMF, cyclophosphamide, methotrexate, 5-fluorouracil; FAC, 5-fluorouracil, adriamycin, cyclophosphamide; CAX, cyclophosphamide, adriamycin, xeloda; NS, not significant*.

Formalin-fixed, paraffin-embedded (FFPE) samples of breast tumors were used for morphological (*n* = 135), immunohistochemical (*n* = 135), and immunofluorescence analyses (*n* = 25).

The procedures followed in this study were in accordance with the Helsinki Declaration (1964, amended in 1975 and 1983). All patients signed informed consent for voluntary participation. The study was approved by the review board of the Cancer Research Institute, Tomsk NRMC on 17 June 2016 (the approval number is 8).

### Morphological Analysis

**Figure 1:**
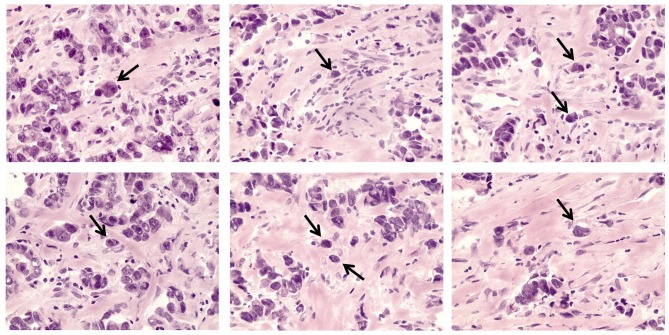
eSTCs in invasive breast carcinoma of no special type. Arrows indicate eSTCs located out of multicellular structures. 400× magnification.

The morphological analysis included the determination of STCs in breast tumors (Figure 1). Five-micrometer-thick hematoxylin and eosin (H&E)-stained sections of FFPE samples were used for the STC analysis using an Axio Lab.A1 light microscope (Carl Zeiss, Germany). STCs (or detached individual tumor cells) were determined in the entire tumor tissue in contrast to tumor buds residing in the invasive front and defined as tumor cells located out of multicellular tumor structures (tubular, alveolar, solid, and trabecular) but similar to them in cytological features. STCs with epithelial morphology (eSTCs) had eosin-stained cytoplasm of different volumes and were larger than immune and stromal cells (tumor cell nuclei ≥3 × the size of lymphocyte). Tumor cells similar to fibroblasts/myofibroblasts or mononuclear leukocytes (lymphoid cells, macrophages) in shape and size were not possible to identify in H&E-stained sections and were revealed using the epithelial marker, cytokeratin 7 (CK7). We attributed these cells to STCs with mesenchymal morphology (mSTCs).

### Immunohistochemical Analysis

Immunohistochemistry was used to assess the expression of estrogen and progesterone receptors (ER and PR), HER2, and Ki-67 in breast tumors using the following antibodies: mouse anti-ER (Dako, Cat. # IR084, clone 1D5, RTU), mouse anti-PR (Dako, Cat. # IR068, clone PgR636, RTU), rabbit anti-HER2 (Dako, Cat. # A0485, 1:800), and mouse anti-Ki-67 (Dako, Cat. # IR626, clone MIB-1, RTU). Immunohistochemistry was performed as previously described (11). ER and PR immunostaining was scored using ASCO/CAP Recommendations (12). HER2 immunostaining was scored using St. Gallen recommendations (13). Ki-67 immunostaining was expressed as the percentage of positively stained cells. At least 10 fields of view and at least 1,000 cells at 400× magnification (field area = 0.196 mm^2^) were analyzed per sample. Molecular subtypes of the IC NST were categorized according to St. Gallen recommendations (13): luminal A (ER^+^ and/or PR^+^, HER2^−^, and Ki-67 < 20%), luminal B (ER^+^ and/or PR^+^, HER2^−/+^, and Ki-67 ≥ 20%), HER2-positive (ER^−^, PR^−^, and HER2^+^), and triple-negative (ER^−^, PR^−^, and HER2^−^).

### Immunofluorescence Analysis

Immunofluorescence staining was used to analyze the morphological and phenotypical heterogeneity of STCs. Seven-micrometer-thick sections were prepared from FFPE tumor samples (*n* = 25), deparaffinized, rehydrated, processed for heat-induced epitope retrieval in PT Link (Dako, Denmark) with high pH buffer, and blocked with 3% bovine serum albumin (Amresco, USA) in PBS. Subsequently, the sections were incubated with a cocktail of primary antibodies: mouse anti-CD133 (MyBioSource, Cat. # MBS5305439, clone 3F10, 1:800), rabbit anti-Snail/Slug (Abcam ab180714, 1:400), and goat anti-CK7 (Santa Cruze, Cat. # sc-70936, 1:50) or mouse anti-CD133 (MyBioSource, Cat. # MBS5305439, 1:800), rabbit anti-N-cadherin (Abcam ab76057, 1:400), and goat anti-CK7 (Santa Cruze, Cat. # sc-70936, 1:50) followed by incubation with the appropriate secondary antibodies: goat anti-mouse IgG H&L (Alexa Fluor 488, Abcam ab150117, 1:200), goat anti-rabbit IgG H&L (Cy3, Abcam ab6939, 1:200), and donkey anti-goat IgG H&L (Alexa Fluor 647, Abcam ab150135, 1:200). Finally, Vectashield mounting medium (Vector Laboratories, USA) containing DAPI was used to detect nuclei and mount the specimens. The samples were analyzed using an LSM 780 NLO confocal microscope (Carl Zeiss, Germany).

Normal endometrial, liver, and tonsillar tissues were used as a positive control for anti-CD133, N-cadherin, and Snail antibodies, respectively. Human skin fibroblasts were used as a negative control for CD133 and N-cadherin staining. Snail expression was heterogeneous in cells of mammary acini and ducts. Negative control for Snail staining was acinar and ductal cells that did not express this protein.

Using CK7 staining, we identified STCs with distinct epithelial- and mesenchymal-like morphologies. eSTCs had abundant cytoplasm and were larger than immune and stromal cells (tumor cell nuclei ≥3 × the size of the lymphocyte). mSTCs were detected as CK7-positive cells, in which the size and the cytological characteristics were similar to those of immune and stromal cells.

CD133-positive STCs were designated as stem cells. Snail was considered as a marker of early EMT (14), whereas N-cadherin—as a marker of advanced EMT (15, 16). Two parameters were used to evaluate the distribution of cells with stem and EMT phenotypes in eSTCs and mSTCs. First, we assessed how often stem and EMT cells were observed in eSTC- and mSTC-positive patients. Second, we calculated the percentage of stem and EMT cells among eSTCs and mSTCs.

### Statistical Analysis

Statistical analysis was performed using STATISTICA 8.0 for Windows (StatSoft Inc., USA). Normal distribution was tested using the Shapiro–Wilk test. Fisher's exact test was applied to assess differences in the frequency of cell subpopulations both between STCs with various morphologies and different clinicopathological parameters. The Mann–Whitney U-test was applied to analyze differences in the percentage of cell subpopulations between STCs with various morphologies. MFS and RFS were evaluated using the Kaplan–Meier estimator with the log-rank test. Differences were considered significant at *p* < 0.05. Differences at 0.05 > *p* < 0.1 were discussed as non-significant trends. Cox proportional hazard analysis was used to assess the association between eSTCs and MFS and RFS. Associations were reported as hazard ratios (HRs) with 95% confidence intervals (95% CIs) and *p*-values (likelihood ratio test).

## Results and Discussion

### Frequency of eSTCs in Breast Cancer: an Association With Clinicopathological Parameters

Here, we assessed the frequency of STCs in breast tumors (*n* = 135). It must be noted that only eSTCs could be detected in the H&E sections of breast tumors (Figure 1). eSTCs were found in 37.0% (50/135) of the breast tumors. Their frequency did not vary between molecular subtypes of breast cancer: 42.3% (22/52)—luminal A, 38.8% (19/49)—luminal B, 22.7% (5/22)—triple-negative, and 33.3% (4/12)—HER2-positive tumors. The frequency of eSTCs did not depend on the parameters of the patients. However, eSTCs were more frequent in large-sized tumors (2–5 cm) (Table 1).

### Association of eSTCs With Breast Cancer Progression

**Figure 2:**
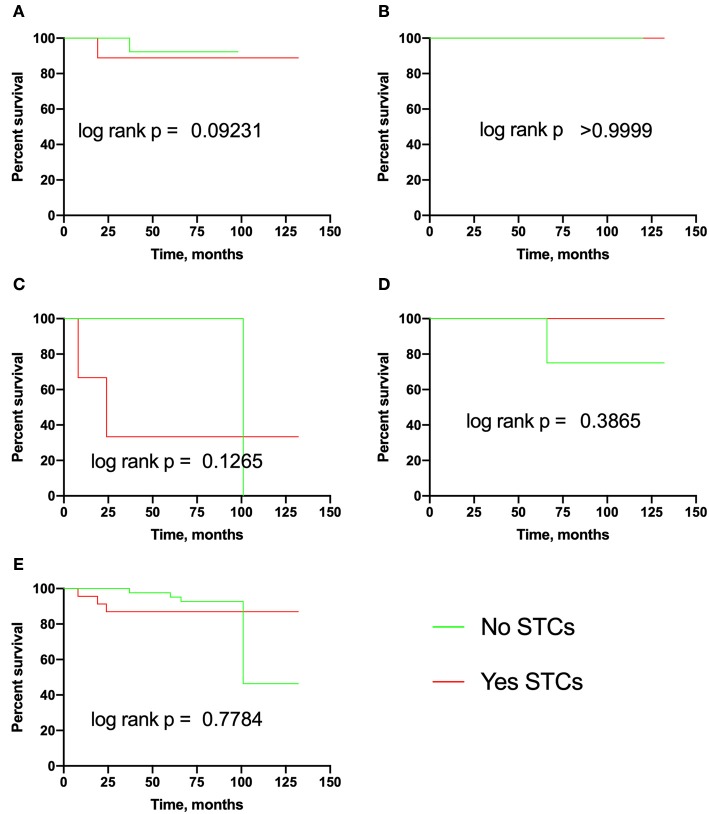
Recurrence-free survival in breast cancer patients with eSTCs. **(A)** Luminal A. **(B)** Luminal B. **(C)** Triple-negative. **(D)** HER2-positive. **(E)** Total group.

In this section, we assessed the association of eSTCs with recurrence, lymph node, and distant metastasis in breast cancer. It turned out that the probability of recurrence and RFS did not depend on eSTCs (Table 2, Figure 2).

**Table 2:** Frequency of recurrences in breast cancer patients with eSTCs.

	**No eSTCs**	**Yes eSTCs**	***P*-values**
Luminal A	3.33 (1/30)	4.54 (1/22)	1.000
Luminal B	0.00 (0/30)	0.00 (0/19)	1.000
Triple-negative	5.88 (1/17)	4.00 (2/5)	0.116
HER2-positive	12.50 (1/8)	0.00 (0/4)	1.000

*P-values indicate differences between patients with the absence (“No”) and presence (“Yes”) of eSTCs*.

By contrast, lymph node involvement was more frequent in eSTC-positive than in eSTC-negative patients [60.0% (30/50) vs. 32.1% (27/84), *p* = 0.002]. This association was significant only in luminal A [59.1% (13/22) vs. 23.3% (7/30), *p* = 0.011]. In luminal B [63.1% (12/19) vs. 44.8% (13/29), *p* = 0.250], triple-negative [60.0% (3/5) vs. 29.4% (5/17), *p* = 0.308], and HER2-positive cancer [50.0% (2/4) vs. 25.0% (2/8), *p* = 0.547], lymph node metastasis was not associated with eSTCs.

The frequency of distant metastasis was also higher in eSTC-positive than in eSTC-negative patients [28.0% (14/50) vs. 9.4% (8/85), *p* = 0.007]. This association was at a borderline significance in HER2-positive [75.0% (3/4) vs. 12.5% (1/8), *p* = 0.066] cancer and not significant in luminal A [22.7% (5/22) vs. 10.0% (3/30), *p* = 0.259], luminal B [21.1% (4/19) vs. 6.7% (2/30), *p* = 0.189], and triple-negative [40.0% (2/5) vs. 11.8% (2/17), *p* = 0.209] cancers.

**Figure 3:**
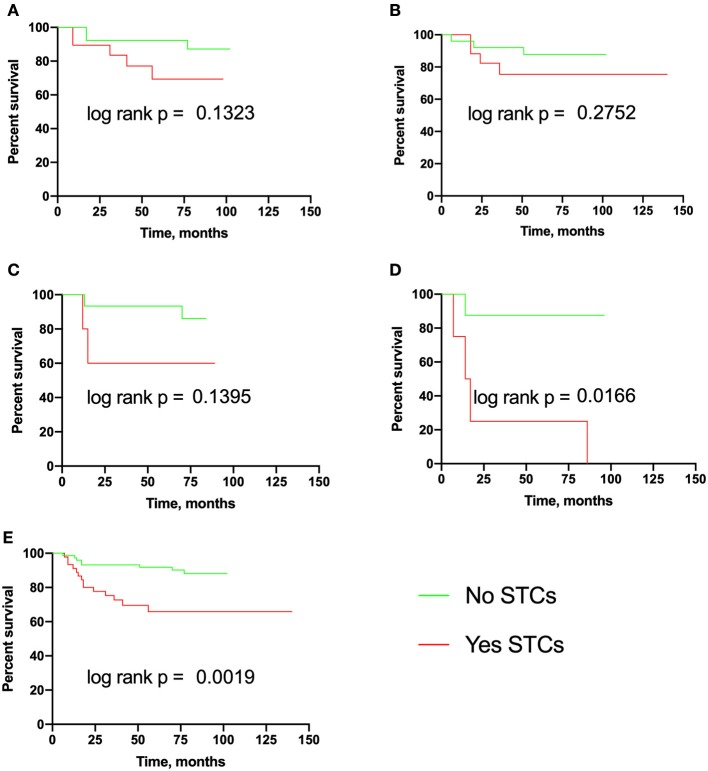
Metastasis-free survival in breast cancer patients with eSTCs. **(A)** Luminal A. **(B)** Luminal B. **(C)** Triple-negative. **(D)** HER2-positive. **(E)** Total group.

The median MFS was not reached in eSTC-positive and eSTC-negative patients both in the total group and in any molecular subtypes (Figure 3). eSTC-positive patients had a higher risk of breast cancer metastasis (HR 3.57, 95% CI: 1.46–8.71; *p* = 0.001). When tumor types were analyzed separately, a higher risk of breast cancer metastasis occurred only in HER2-positive patients (HR 8.49, 95% CI: 1.29–55.59; *p* = 0.016).

### Morphological Heterogeneity of STCs

**Figure 4:**
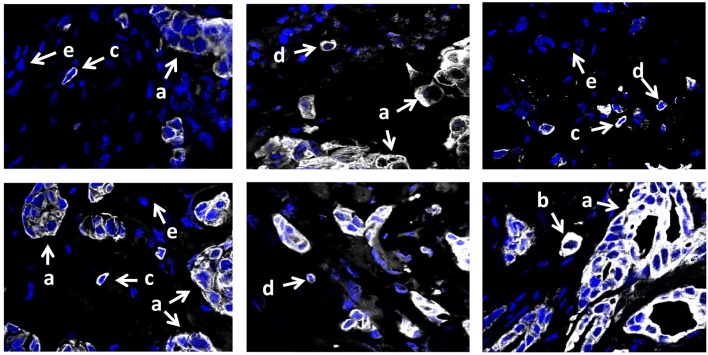
CK7 expression in invasive breast carcinoma of no special type. Multicellular structures are formed by tumor cells with epithelial morphology (marked by the letter “a”). Few CK7-positive STCs have epithelial morphology and do not differ from tumor cells of multicellular structures (marked by the letter “b”). Numerous CK7-positive mSTCs with fibroblast- (marked by the letter “c”) or lymphocyte-like (marked by the letter “d”) morphology are similar to CK7-negative stromal cells (marked by the letter “e”). 400× magnification.

To assess the morphological heterogeneity in STCs, we analyzed the expression of the epithelial marker, cytokeratin 7 (CK7), in 15 breast cancers with eSTCs and 10 cases without these cells. CK7-positive STCs were represented by cells with both distinct epithelial and mesenchymal (fibroblast- or lymphocyte-like) morphologies (Figure 4). In particular, 40.0% (10/25) of the cases had eSTCs, 28.0% (7/25) of the cases had mSTCs, and 32.0% (8/25) of the cases had eSTCs and mSTCs simultaneously.

CK7-positive STCs tended to be observed more frequently in luminal B cancers, whereas CK7-negative STCs were more often in triple-negative cancers (Table 3). In HER2-positive cancer, the frequencies of CK7^+^ and CK7^−^ STCs were not compared due to small patient numbers in each group (Table 3).

**Table 3:** Frequency of eSTCs and mSTCs in different molecular subtypes of breast cancer.

**Patient groups**		**eSTC+mSTC–**	**eSTC–mSTC+**	**eSTC+mSTC+**
Luminal A	1	20.0 (2/10)	30.0 (3/10)	50.0 (5/10)
Luminal B	2	66.7 (6/9) p_2−1_ = 0.069 p_2−3_ = 0.181	11.1 (1/9)	22.2 (2/9)
Triple-negative	3	0.0 (0/3)	66.7 (2/3) p_3−2_ = 0.127	33.3 (1/3)

*Fisher's exact test. p_2−1_, p_2−3_, and p_3−2_, differences between luminal B (2), luminal A (1), and triple-negative (3) cancers*.

Morphological and immunofluorescence analysis showed a high level of concordance (88%) in the identification of eSTCs. In 12% (3/25) of the cases, eSTCs were not detected morphologically but were observed by immunofluorescence staining with CK7. Most likely, it was related to the scarcity of CK7-positive STCs in H&E sections or their intermediate epithelial–mesenchymal morphology. It must be noted that immunofluorescence analysis not only confirmed the absence of eSTCs in H&E stained sections of some cases but also showed the presence of mSTCs in these cases.

Based on the morphological analysis, we classified breast cancer patients to three groups: with eSTCs (mSTCs−) only, with mSTCs (eSTCs−) only, and with eSTCs and mSTCs (eSTC+mSTC+) simultaneously.

### Heterogeneity of STCs in Stem and EMT Features

**Figure 5:**
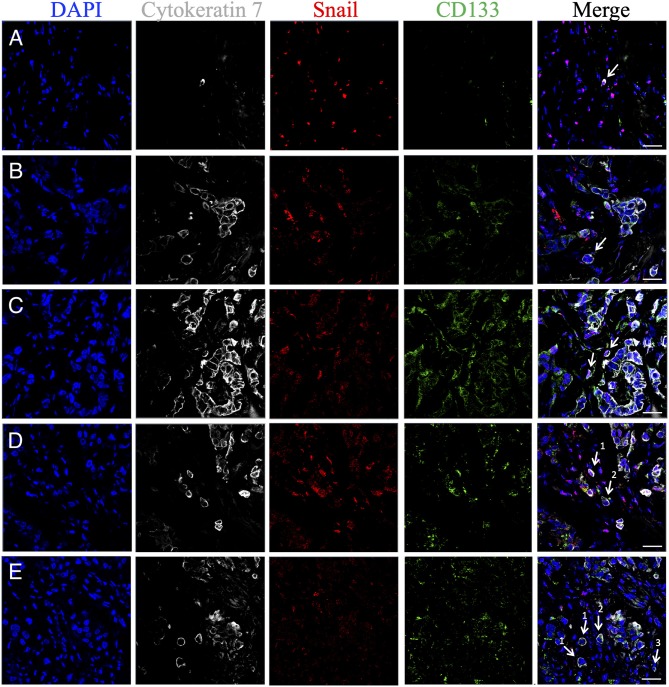
Phenotypic heterogeneity of eSTCs and mSTCs in stem and early EMT features. **(A)** Nonstem mSTC with early EMT (CK7^+^CD133^−^Snail^+^) surrounded by immune and stromal cells. **(B)** eSTC without stem and EMT features (CK7^+^CD133^−^Snail^−^) near the multicellular structure. **(C)** Stem mSTC with early EMT (CK7^+^CD133^+^Snail^+^) near the multicellular structure. **(D)** Stem mSTC with early EMT (CK7^+^CD133^+^Snail^+^) among microenvironment cells (1) and stem eSTC without EMT (CK7^+^CD133^+^Snail^−^) similar in size to tumor cells composing multicellular structures (2). **(E)** Nonstem eSTC without EMT (CK7^+^CD133^−^Snail^−^) (1), stem eSTC without EMT (CK7^+^CD133^+^Snail^−^) (2), and nonstem mSTC without EMT (CK7^+^CD133^−^Snail^−^) among microenvironment cells. eSTCs were identified based on their similarity in size to tumor cells of multicellular structures, whereas mSTCs, to immune/stromal cells. Scale bar, 50 μm.

**Figure 6:**
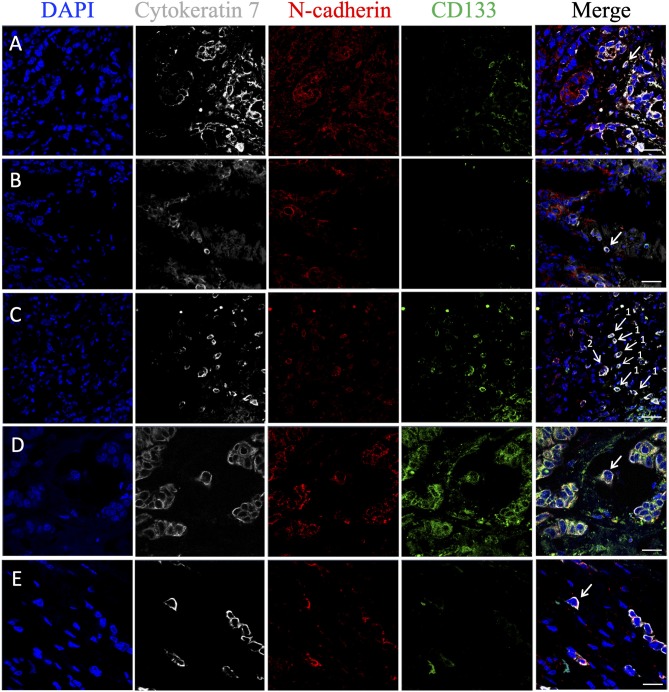
Phenotypic heterogeneity of eSTCs and mSTCs in stem and advanced EMT features. **(A)** Stem mSTC with advanced EMT (CK7^+^CD133^+^N-cadherin^+^) near the multicellular structures. **(B)** mSTC without the stem and EMT features (CK7^+^CD133^−^N-cadherin^−^) among microenvironment cells. **(C)** Stem mSTC with advanced EMT (CK7^+^CD133^+^N-cadherin^+^) (1) and the group of two tumor cells (were not considered as STCs in the study) with stem and advanced EMT features (2) among microenvironment cells. **(D)** Stem eSTC with advanced EMT (CK7^+^CD133^+^N-cadherin^+^) near the multicellular structures. **(E)** Nonstem eSTC with advanced EMT (CK7^+^CD133^−^N-cadherin^+^) near the multicellular structures. Scale bar, 50 μm **(A–C)** and 20 μm **(D,E)**.

Markers for stemness (CD133) and EMT (Snail and/or N-cadherin) were assessed in eSTCs and mSTCs (Table 4, Figures 5, 6). However, the frequencies of tumors with stem and/or EMT cells did not differ between patient groups, eSTC (mSTC–) and mSTC (eSTC–) (Table 4). Nevertheless, the differences in the percentages of different subpopulations were observed among eSTCs and mSTCs (Table 5).

**Table 4:** Frequency of cells with stem and EMT phenotypes among eSTCs and mSTCs.

**Cells**	**eSTC+mSTC– (1)**	**eSTC–mSTC+ (2)**	**eSTC+mSTC+**	
			**eSTCs (3)**	**mSTCs (4)**
CK7^+^CD133^−^Snail^−^	100 (9/9)	100 (5/5)	100 (7/7)	100 (8/8)
CK7^+^CD133^−^Snail^+^	78 (7/9)	40 (2/5)	86 (6/7)	50 (4/8)
CK7^+^CD133^+^Snail^−^	67 (6/9)	100 (5/5)	43 (3/7)	0 (0/8) p_3−4_ = 0.076 p_2−4_ = 0.0008
CK7^+^CD133^+^Snail^+^	89 (8/9)	60 (3/5)	71 (5/7)	25 (2/8)
CK7^+^CD133^−^N-cadherin^−^	100 (9/9)	100 (5/5)	100 (7/7)	88 (7/8)
CK7^+^CD133^−^N-cadherin^+^	89 (8/9)	100 (5/5)	86 (6/7)	38 (3/8)
CK7^+^CD133^+^N-cadherin^−^	56 (5/9)	40 (2/5)	71 (5/7)	13 (1/8) p_3−4_ = 0.040
CK7^+^CD133^+^N-cadherin^+^	100 (9/9)	60 (3/5)	57 (4/7)	25 (2/8)

*Fisher's exact test. p_2−4_ and p_3−4_, differences between groups of patients with only mSTCs (2) and simultaneously with eSTCs (3) and mSTCs (4)*.

**Table 5:** Percentage of cells with stem and EMT phenotypes among eSTCs and mSTCs.

	**eSTC+mSTC– (1)**	**eSTC–mSTC+ (2)**	**eSTC+mSTC+**	
			**eSTCs (3)**	**mSTCs (4)**
CK7^+^CD133^−^Snail^−^	70.70 (24.20–80.00)	88.10 (80.00–94.00) p_1−2_ = 0.031	62.70 (46.50–78.20)	73.55 (51.30–100.00)
CK7^+^CD133^−^Snail^+^	5.00 (1.70–7.50)	0.00 (0.00–1.40)	16.70 (6.40–24.10)	7.70 (0.00–37.45)
CK7^+^CD133^+^Snail^−^	6.10 (0.00–19.00)	3.40 (2.00–4.50)	0.00 (0.00–8.60)	0.00
CK7^+^CD133^+^Snail^+^	12.30 (9.40–29.30)	2.40 (0.00–8.50) p_1−2_ = 0.025	15.38 (0.00–21.60)	0.00 (0.00–3.20)
CK7^+^CD133^−^N-cadherin^−^	50.00 (17.20–73.20)	68.30 (50.0–78.10)	64.30 (51.0–66.70)	95.45 (67.85–100.00)
CK7^+^CD133^−^N-cadherin^+^	17.20 (15.60–33.30)	16.40 (7.30–22.70)	20.80 (13.60–33.30)	0.00 (0.00–13.65)
CK7^+^CD133^+^N-cadherin^−^	1.20 (0.00–10.30)	1.20 (0.00–3.90)	6.60 (0.00–21.40)	0.00
CK7^+^CD133^+^N-cadherin^+^	16.70 (7.70–21.70)	7.40 (0.00–12.20) p_1−2_ = 0.011	3.40 (0.00–17.70)	0.00 (0.00–3.05)

*Percentages are given as medians and quartiles: Me (Q1–Q3). Mann–Whitney U-test. p_1−2_ and p_3−4_, differences between groups of patients with eSTCs (1) only, with mSTCs (2) only, and with eSTCs (3) and mSTCs (4) simultaneously*.

Stem cells with early (CK7^+^CD133^+^Snail^+^) and late (CK7^+^CD133^+^N-cadherin^+^) EMT were lower in the mSTC (eSTC–) group compared to the eSTC (mSTC–) group (Table 5). In the patient group with the simultaneous presence of eSTCs and mSTCs, CK7^+^CD133^+^Snail^−^ and CK7^+^CD133^+^N-cadherin^−^ cells tended to be rare among mSTCs compared to eSTCs (Table 4). Surprisingly, the percentage of these stem-like non-EMT cells did not differ between eSTCs and mSTCs in patients in which the tumor simultaneously contained eSTCs and mSTCs (Table 5).

Non-stem and non-EMT (CK7^+^CD133^−^Snail^−^) cells were predominant among eSTCs and mSTCs in all three groups of patients: eSTC (mSTC–), mSTC (eSTC–), and eSTC+mSTC+ (Tables 4, 5).

## Discussion

STCs, i.e., detached individual tumor cells, are widely recognized by pathologists in H&E-stained sections, but their phenotypic features and role in cancer progression remain to be elucidated. Some studies reported genetic analysis of STCs, but in many cases, these were not detached individual cells and were obtained from tumor samples by mechanical dissociation (17) or from multicellular structures by laser microdissection (18). Other studies described STCs at the invasive front, for example, in tumor budding (4), and investigated their genomic copy number profiles (19).

In this study, we determined STCs in the entire tumor tissue as cells with epithelial- or mesenchymal-like morphology that were represented by subpopulations with various EMT and stem phenotypes. However, only eSTCs were associated with breast cancer metastasis. In addition, eSTCs were prevalent in large-sized breast tumors. This finding may explain why large breast tumors metastasize more often than small tumors (20). STCs probably appear in the tumor by detaching from multicellular tumor structures. Most likely, it may occur through two different mechanisms. The first mechanism, demonstrated by various studies, is that STCs are a result of detaching of leader cells at the invasive edge of multicellular tumor structures (21). The second mechanism is rather hypothetical and may be due to the pushing of tumor cells from multicellular structures to the stroma. As suggested by Rosenblatt and coauthors, such basal extrusion occurs in conditions of disrupting the S1P-S1P2 signaling pathway underlying physiologically normal apical extrusion of cells that completed their life cycle (22, 23).

It is reasonable to assume that detaching cells from multicellular structures, or collective-individual transition, should be accompanied by EMT and following the inhibition of cell–cell adhesion. However, our results show that most STCs are not characterized by the expression of Snail or N-cadherin. This can be explained either by the fact that basal extrusion, if it occurs, is not related to EMT or by EMT reversibility (i.e., mesenchymal–epithelial transition, MET). In fact, the study of EMT in cancers resulted in the understanding of this process as a consecutive spectrum of cell states from initial epithelial through intermediate hybrid or metastable to terminal mesenchymal phenotypes (24, 25).

The term “epithelial–mesenchymal plasticity,” which is more and more often used at the present time, most accurately describes the EMT–MET interconversion, with the possibility of phenotypic changes from epithelial to mesenchymal states and vice versa with a stop at any stage of the process (25, 26). The presence of cells with varying degrees of EMT among STCs with epithelial and mesenchymal morphology most likely reflects epithelial–mesenchymal plasticity.

At present, it is known that complete EMT is less effective for cancer progression than partial EMT that retains the molecular and morphological features of epithelial cells (26). Tumor cells with a hybrid epithelial–mesenchymal phenotype are more adaptive to the tumor microenvironment and resistant to immune reactions and demonstrate a pronounced colony-forming ability (27). In our study, eSTCs expressing Snail or N-cadherin most likely possess a hybrid (metastable) phenotype. This can explain the significant association between eSTCs and high probability of breast cancer metastasis. In addition, our results indicate that the presence of EMT features, particularly Snail or N-cadherin expression, is not accompanied by an obligatory transition from epithelial to fibroblast- or lymphocyte-like cell shape. The absence of spindle-like shape in tumor cells undergoing EMT was reported previously (28).

Nevertheless, tumor cells with mesenchymal morphology can be identified in H&E sections if they are located in multicellular structures together with epithelial-like cells. For example, the three-dimensional reconstruction of tumor tissue sections showed that tumor cells located at the invasive front of the collective invading structure rarely have spindle-like or round (mesenchymal) shape (29). In histological specimens, it is almost impossible to observe STCs with fibroblast- or lymphocyte-like morphology without epithelial markers. This fact should be considered when a pathologic response to neoadjuvant chemotherapy is assessed. It is well-known that a pathologic complete response (pCR) is a favorable prognostic factor. However, because pCR is determined by a pathologist based on the assessment of H&E sections, STCs with fibroblast- or lymphocyte-like morphology cannot be detected and the diagnosis can be inaccurate. Despite a high probability of this mistake, pCR remains a marker of good prognosis. Does it mean that mSTCs are not significant for cancer progression? The association between eSTCs and high frequency of breast cancer metastasis probably confirms the low importance of mSTCs in the formation of metastases. In reality, tumor cells with fibroblast-like shape were found to have a decreased aggressiveness (30). However, future studies should clarify the significance of eSTCs and mSTCs in metastasis.

According to our study, some eSTCs and mSTCs demonstrated features of either stemness, EMT, or simultaneous stemness and EMT that make them similar to circulating tumor cells (CTCs). For example, breast CTCs were found to express both epithelial and mesenchymal markers demonstrating EMT features (31). We also showed that CTCs are highly heterogeneous population in breast cancer and have similar phenotypes to STCs: various combinations of the stem and EMT features or the absence of these marks (32). The similarity of STC and CTC phenotypes may indicate the high ability of STCs to intravasation (33).

The relationship between stemness and EMT in tumor cells is widely discussed (34); however, opinions about causal relationships between these processes are contradictory. The recent study showed that EMT inhibition results in the acquisition of stemness and the initiation of breast cancer metastasis. In contrast, EMT activation suppressed stem features (27). These findings are in agreement with our results that eSTCs are associated with breast cancer metastasis. Moreover, it was found that fibroblast-like cells with EMT features partially maintain the polarity, attach tightly to the extracellular matrix, and remain quiescent. It was assumed that these cells may irreversibly transform to cancer-associated fibroblasts (35).

## Conclusions

STCs demonstrate morphological diversity and phenotypical heterogeneity in stem and EMT features. STCs with epithelial morphology are associated with breast cancer metastasis and probably demonstrate a hybrid (metastable) EMT phenotype. Given these findings, eSTCs represent an attractive object in the study of mechanisms and key features that are typical of metastatic “seeds.” In general, the determination of eSTCs in histological sections of breast tumors may be considered as an available prognostic marker of metastasis.

## Data Availability Statement

The datasets generated for this study are available on request to the corresponding author.

## Ethics Statement

The studies involving human participants were reviewed and approved by Ethic Committee of the Cancer Research Institute, Tomsk NRMC (the approval number is 8, on 17 June 2016). The patients/participants provided their written informed consent to participate in this study.

## Author Contributions

VP and MZ designed the research. MZ performed histology image analysis. LT, OS, and EK performed immunofluorescence analysis, score, and analysis of STCs in breast cancer samples. LT prepared confocal images. LT and TG performed data analysis. LT, ED, and VP drafted the work. VP supervised the study and the final approval of the version to be published.

### Conflict of Interest

The authors declare that the research was conducted in the absence of any commercial or financial relationships that could be construed as a potential conflict of interest.
